# Effect of the Process Parameters on the Adhesive Strength of Dissimilar Polymers Obtained by Multicomponent Injection Molding

**DOI:** 10.3390/polym13071039

**Published:** 2021-03-26

**Authors:** Luciano Pisanu, Leonardo Costa Santiago, Josiane Dantas Viana Barbosa, Valter Estevão Beal, Marcio Luis Ferreira Nascimento

**Affiliations:** 1 SENAI CIMATEC, Institute of Innovation for Forming and Joining of Materials, Av. Orlando Gomes 1845, Piatã, Salvador 41650-010, Brazil; lecosant@gmail.com (L.C.S.); josianedantas@fieb.org.br (J.D.V.B.); valtereb@fieb.org.br (V.E.B.); 2 Nanotechnology Group, Graduate Program in Industrial Engineering, Polytechnic School, Universidade Federal University of Bahia, R. Aristides Novis 2, 6° Andar, Federação, Salvador 40170-115, Brazil; mlfn@ufba.br

**Keywords:** wood composites, plastics, lap shear, hybrid joints

## Abstract

The growing demand in the consumer market for products with sustainable technologies has motivated new applications using overmolded natural fiber composites. Therefore, studies have been conducted mainly to understand the adhesive properties of overmolded parts. In the present study, a polypropylene (PP) composite with 30% coconut fibers without additives was developed with the aid of a corotating twin screw extruder. Subsequently, a multicomponent injection mold was developed based on the geometry of the ISO 527 type I specimen, in which samples overmolded with PP and PP–coconut-fiber composite, with the overlap in the central area, were obtained to evaluate the adhesive strength of dissimilar materials. The objective of this study was to evaluate the bond between PP and PP–coconut-fiber composite under different processing conditions using an adhesive strength testing device to perform a pure shear analysis. The experimental conditions followed a statistical design considering four factors in two levels and a significance level of 5%. The results indicated that adhesive strength increased significantly as the overlap area increased. It was observed that temperature and injection flow rate were the factors that most contributed to strengthening the bonds of dissimilar materials.

## 1. Introduction

According to Estácio [1], injection molding is one of the most important industrial processes for obtaining large-scale plastic products. The process essentially consists of fusing the polymer inside a heated cylinder and injecting the polymer under pressure inside a mold, where it cools quickly and adopts its final shape. To ensure the quality and reproducibility of injected parts, the injection molding machine offers a set of options that can guarantee the quality and reproducibility of the mechanical and dimensional properties and prevent the emergence of defects, such as shrink cavities, warping, and bubbles, among others, as explained by Cavalheiro [2] and Peixoto [3].

From history, the American inventors John Wesley Hyatt (1837–1920, Figure 1) and his brother Isaiah Smith Hyatt (?–1885) patented the first injection molding machine in 1872 (US patent 133,229). John also invented celluloid in 1869 (US patent 50,359). This molding machine was relatively simple compared to modern equipment because it worked like a large hypodermic needle using a plunger to inject plastic through a heated cylinder into a mold.

The increasing use of polymeric materials has culminated in an increase in functionality requirements, leading designers to combine materials in the same component to produce so-called multimaterials, as described by Wargnier [4]. In this sense, the multicomponent injection process provides easy fabrication and significantly simplifies the assembly operations used to manufacture parts associated with more than one polymeric material, as described by Banerjee et al. [5]. Another great advantage is due to the possibility of combining several characteristics into the same component, thereby increasing the component functionality by combining the properties of mechanical strength and touch, damping against vibrations or impacts, and incorporating aesthetic factors, such as gloss and roughness, as noted by Nguyen et al. [6].

However, the rapid development of new materials, including composites, has resulted in difficulties in analyzing the interactions between the components when the two components are rigid. According to Kraus et al. [7], the good performance of the injection molding of dissimilar materials is attributed to the strong interfacial adhesion generated from the interactions between polymers.

Adding natural fibers to reinforce composites presents challenges due to wide variation in the properties and characteristics of the fibers, especially in multicomponent injection molding, where it is necessary to ensure the materials bond to guarantee the functionality of the part. Fibers from coconut generally have good tensile strength, high modulus of elasticity, high humidity, and good degradability [8]. Cellulose is the main structural component of such plant fibers. Machado et al. [8] analyzed the shrinkage of coconut fibers from present work under heating, compared with traditional thermal analysis under the same heating treatments.

During the injection process of polymer composites, rheological behavior shows that the orientation of the fibers in each layer varies from center region to walls. Fibers were oriented parallel to the wall and were randomly oriented in the central region. This orientation promoted an anisotropic elastic modulus under stress and bending, as Patcharaphun [9] noted in his work. According to Karthikeyan [10], the upper layer, called skin, has a thickness between 15–20% of the total thickness and was created by rapid cooling, generating extensional deformation during injection flow. Fibers in this thin layer were oriented parallel to the cavity wall, and fibers located in the center had a total thickness of 60–70%. In the center, we observed structures that were less oriented along the flow direction, as explained by Karthikeyan [10]. The orientation of the fibers in the center could be determined by the flow gradient and the position and size of the material inlet. At low shear rates, fibers promoted considerable resistance to flow, and at high shear rates, the flow was almost constant, resulting in the formation of larger oriented layers, as shown in the studies done by Akai and Barkley [11] and Bright et al. [12].

In multicomponent injection molding, the process variables are very important to achieve a good finished surface and can alter the adhesive properties of dissimilar materials.

Several methods are used to test the adhesive strength of polymeric materials, and a commonly used standard for evaluating their adhesive strength is the T-peel test performed according to the ASTM D1876 [13]. Therefore, some researchers, such as Li et al. [14], proposed several techniques for reducing peel and interfacial stresses, including a spew fillet, adhesive thickness, mixed adhesive and, tapered plate with different shapes, different thicknesses, adherent widths, and tapered length and thickness, etc. When the polymers are rigid and planar, the lap shear test (Strength of Adhesively Bonded Rigid Plastic Lap-Shear Joints in Shear by Tension Loading-ASTM D3163-01) [15] could be adopted.

However, all of the abovementioned tests focus on measuring the force required by two separate surfaces. These tests do not assess the pure shear stress caused by the presence of other forces similar to those generated by the bending moment because the single lap joint geometry favors this event, as Bamberg et al. [16] mentioned in earlier research.

In this work, to evaluate the adhesive strength of dissimilar polymers and composites of coconut fibers, a specific procedure was applied considering a Brazilian patent on a new mechanical device, as described previously by Pisanu et al. [17] (BR 10 20,160 21054). This apparatus measures the adhesive strength between two overmolded materials in an overlapping region and disregards the influence of the forces resulting from the bending moment and/or peeling stress, as described in recent studies performed by Pisanu et al. [18,19,20]. These experiments evaluated the influence of the injection molding variables over the adhesive forces of overmolded polypropylene (PP) and PP–coir fiber composites, as described in [20]. It is expected to observe good adhesion because PP polymer chains are the same at interface.

## 2. Materials and Methods

In this study, a factorial design experiment was used, in which 4 input factors, the injection temperature, injection flow rate, holding pressure, and overlap region, were applied in two levels, resulting in a total of 16 experimental treatments for molding the specimens, and 8 specimens were injected for each condition (replication).

**Figure 2:**
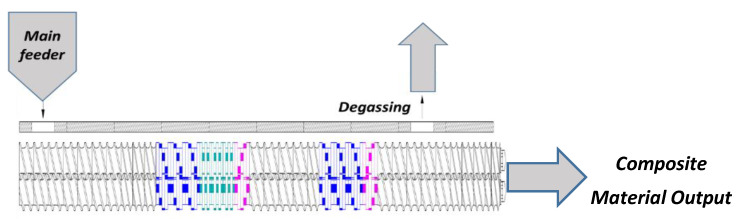
Screw profile scheme used in the present experiment.

The materials used in this experiment were the Braskem EP 440 L heterophasic PP copolymer and dry and prewashed coconut fibers from Frysk Industrial, Aurantiaca Group, Bahia, Brazil. In the formulation of the composite with 30% coconut fibers, the fibers were first dried in a circulating oven at 90 ± 5 °C for 24 h, and, after weighing, a premixture was combined for 2 min in an automatic mixer. The compositions were then measured at the main feed point of a corotating twin screw extruder manufactured by Imacom SP, Brazil (model DRC, 30:40 IF with a screw diameter of 30 mm and L/D ratio of 40). The conditions used to process these composites were as follows: a screw speed of 140 rpm, a feed speed of 8 rpm, a mass temperature of 184 °C, a temperature profile of Z_1_ = 155 °C, Z_2_ = 160 °C, Z_3_ = 165 °C, Z_4_ − Z_7_ = 170 °C, Z_8_ − Z_10_ = 190 °C, and a water tank temperature of Z_11_ = 32 °C. The screw profile used is composed of two mixing zones formed by kneading blocks of 45° and 90°, and the other elements are intended for material transport. Figure 2 shows the screw profile used in this experiment.

The overmolded samples were injected in an Arburg Allrounder 370 S made in Germany, multicomponent injector with 70 tons of closing force and two injection units: 1 horizontal unit with a 35 mm plasticizing screw and 1 vertical unit at 90° with a 30 mm plasticizing screw.

For this test, the composite was injected into Injection Unit 1 with the previously established fixed parameters, and PP with the changing variables, as shown in Table 1, was injected into Injection Unit 2.

This experimental design was chosen because it could be used to evaluate which injection and overlap parameters have a greater effect on the adhesive strength of dissimilar polymers, that is, the adhesion between PP and the coconut fiber composite (PPFC). More details are presented in [20], including moment and peeling issues.

**Figure 3:**
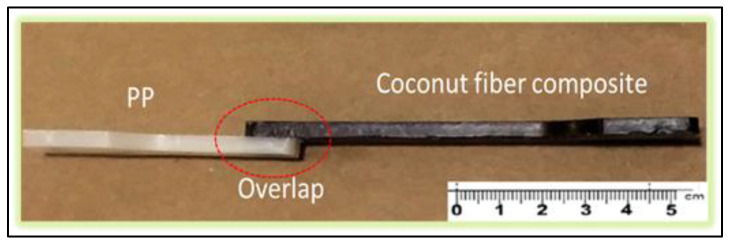
Demonstration of the overlap between the materials.

Overmolded samples, as shown in Figure 3, were produced for each of the 16 outlined levels.

**Figure 4:**
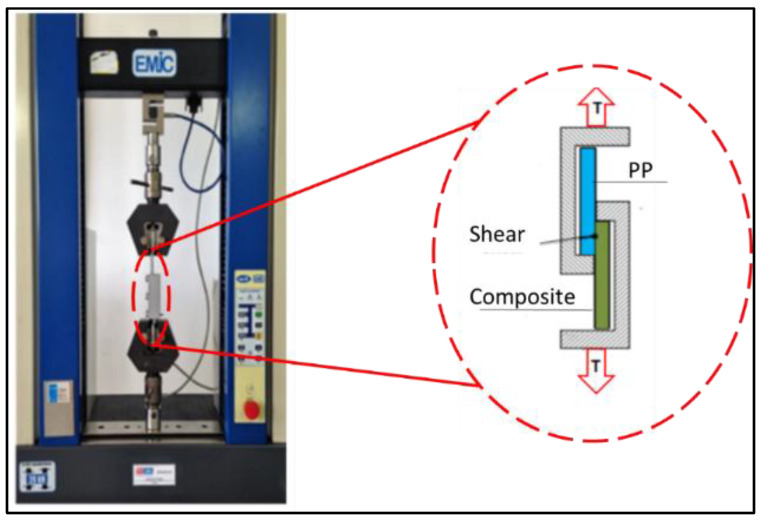
Schematic of the shear stress adapted to a universal testing machine.

Samples were stored for 24 h at a temperature of 23 °C, relative humidity of 55%, and, after being submitted, were cut at the ends; the central region was approximately 60 mm, including the overlap region. Then eight samples were hand-placed into the device shown in Figure 4 and submitted to a tension load on an INSTRON Model 8872 machine at a rate of 1 mm/min.

In this experimental study, the Minitab 18 software was used, and the *p*-values (significance) obtained from an ANOVA with a 95% confidence interval were presented by considering the different test responses. The importance of each factor and its interaction was verified based on analysis of variance (ANOVA). The present study assumed (hypothesized) that the variables of holding pressure (HP), process temperature (T), injection flow rate (Fr), and overlap (O) significantly affect the adhesive strength of the materials. The proposals that explored the factorial technique directly addressed the adhesive strength between dissimilar materials by longitudinal overlapping. The width of the specimen was 10 mm and the overlaps were 12 and 16 mm, combining the other injection variables as temperature, holding pressure, and injection flow rate. Table 2 presents the results and all the variables analyzed in this experiment.

To understand the influences on the overlap area only, an experiment was designed with overlap samples of 12, 16, and 22 mm preserving the same injection conditions in Injection Unit 1 and all the lower level conditions (L) for Injection Unit 2, cited in Table 1.

## 3. Results and Discussions

**Figure 5:**
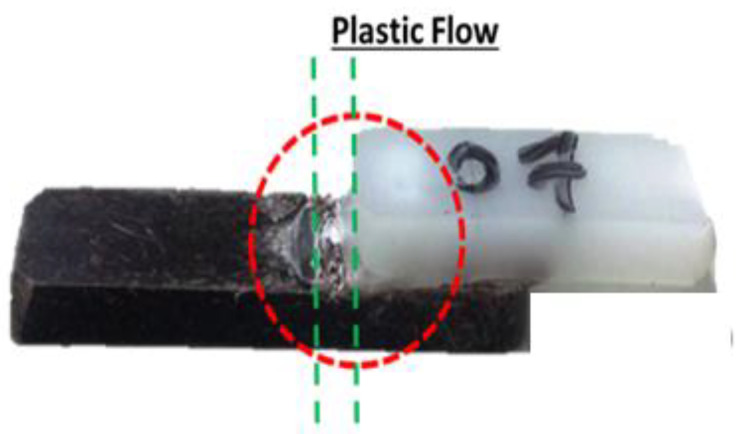
Flow between polymeric joints.

In Figure 5 below is presented the sample tension through the device. It can be observed that there was a flow between the overlapping joints that induced shear stresses. 

Table 3 shows the ANOVA results related to the process temperature, injection flow rate, and holding pressure for variable overlaps of 12 and 16 mm. As results, it presents the degrees of freedom (DF), the sum of squares (SS), and the mean square (MS) used to calculate the *F* distribution (Fisher–Snedecor distribution), in addition to the corresponding *p*-value observed in this study.

**Figure 6:**
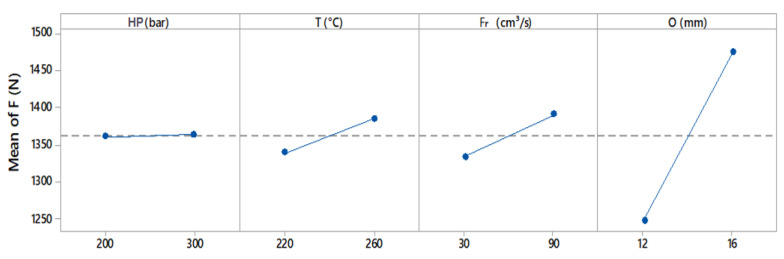
Main factors contributing to strength (N), taking into account the overlaps of 12 and 16 mm.

In Figure 6, the graph of the adjusted mean of the main effects assists in evaluating the effect of the macrovariables acting alone on the counterpart variables. In this analysis, except for the holding pressure, all the factors contributed significantly to the adhesive strength of dissimilar materials, following a linear trend. On the *y*-axis, it is possible to observe the maximum shear force attributed to each variable independently.

It is possible to observe that the overlap had a greater influence than other factors on strength, as expected. A larger contact surface area can increase the occurrence of mechanical anchoring and chain interlocking, which are the main adhesion mechanisms of compatible polymers. Holding pressure exerted a less significant influence on the adhesive strength. From factorial analysis, Equation (1) of the multiple linear regression is obtained, involving parameters presented in Table 3:

Strength (N) = *b*_0_ + *b*_1_ HP + *b*_2_ T + *b*_3_ Fr + *b*_4_ O (1)

where Strength (N) is the outcome variable, *b*_0_ is a simple constant, and *b_i_* (*i* = 1 to 4) are the coefficients of the respective predictors (HP, T, Fr, and O). Applying statistics to the present data, they result in Equation (2):

Strength (N) = 221.3 + 0.033 HP + 1.147 T + 0.957 Fr + 57.09 O(2)

where all *b* values are positive, meaning that there is a positive relationship between every predictor (HP, T, Fr, and O) and the outcome (N). Thus, as HP increases, N increases, and so on.

**Figure 7:**
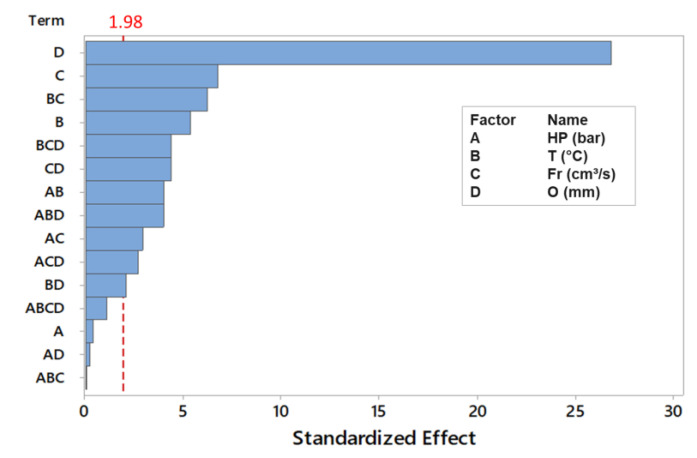
Standardized effects of the process parameters.

Following this analysis, the overlap region is again shown to be the most relevant factor influencing the bonding of the materials (due to its higher coefficient *b*_4_), followed by the process temperature (due to coefficient *b*_2_) and injection flow rate (coefficient *b*_3_), although these factors are less relevant. The design of experiments (DOE) and the Pareto diagram presented in Figure 7 made it possible to evaluate the correlation between all the process variables and the standardized effect of overlapping the bonded materials.

By analyzing the Pareto diagram, we found that, after overlap, the injection flow rate appears to be the second most significant factor, followed by the processing temperature. The holding pressure was only relevant when combined with the injection flow rate and overlap, and otherwise, these factors interacted concomitantly.

Taking into account the injection macrovariables, combining the flow rate and temperature, a positive effect was observed on adhesive strength, and their interaction provided the best results regardless of the proposed overlap.

**Figure 8:**
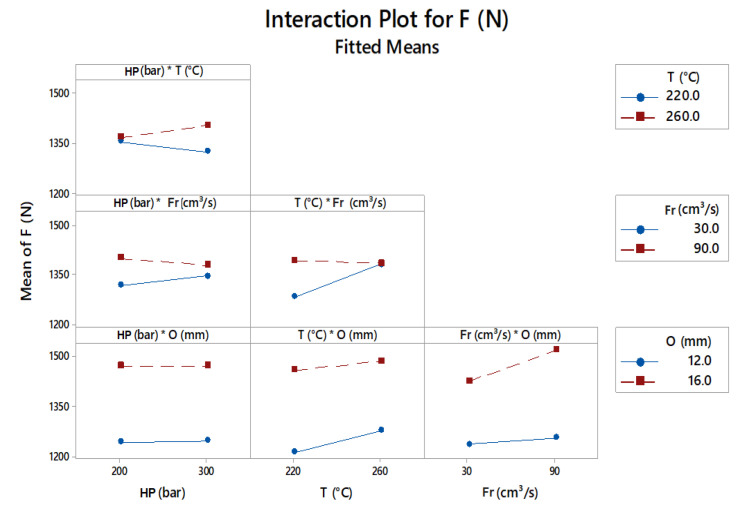
Interactions between factors and the adjusted means.

The final temperature of the molten polymer is due to viscous dissipation heating, a phenomenon that is mainly governed by the filling speed and friction between the sliding layers of fluid, which generates heat, as Tadmor and Gogos [21] and Michaeli and Lindner [22] cited. Based on this hypothesis, it is possible to infer that the thermal conditions programmed in the injection molding process may have been affected by the higher injection flow rate, which explains the strong influence of this particular variable on adhesive strength. According to Peixoto [3], injection speed is a process variable that usually changes the polymer temperature due to viscous dissipation effects, and according to interactions shown in Figure 8, this effect was more significant for the geometry with a 16 mm overlap. However, nonsignificant effects of holding pressure were unexpected because holding pressure compensates the volumetric shrinkage of the molded material and exerted pressure under the material added after the first injection phase.

According to Candal et al. [23], the diffusion of polymeric materials occurs as soon as the molten material wets the substrate. After solidifying the adherent material, an interface with a concentration gradient between dissimilar materials should be obtained. Increasing the holding pressure may increase residual stress at interface, creating a loss in adhesive strength. Combing holding pressure with a higher injection flow rate could contribute negatively to adhesive strength.

Combined with temperature, the increase in injection flow seems to lead to two opposing phenomena: a higher process temperature at the moment of contacting the substrate, favoring interdiffusion and simultaneously increasing the effectiveness of the holding pressure, which results in greater residual stress. The process temperature was proven to be a predominant factor in the adhesion of dissimilar polymers, and this result is supported by other studies of multicomponent injection, as indicated by several studies, such as those by Peixoto [3], Candal et al. [23], Chen et al. [24], Patankar [25], and Raia [26].

**Figure 9:**
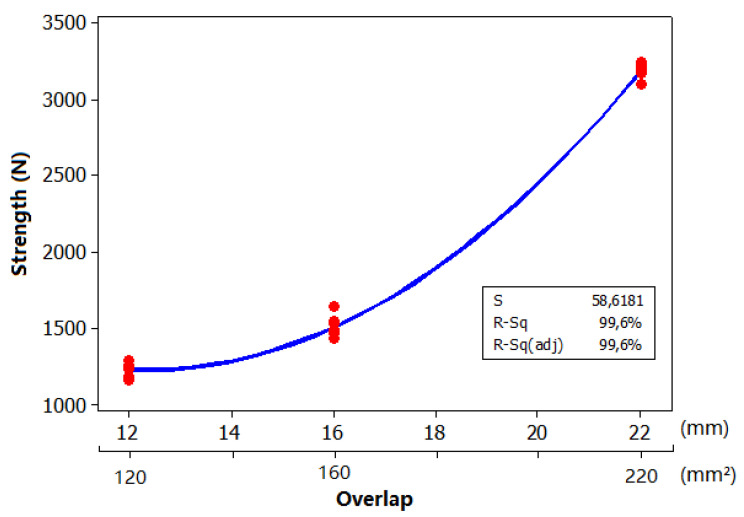
Adhesive strength of the PPCF and PP composite plotted as regression functions of the overlap area (48, 64, and 88 mm^2^).

For an additional experimental test, we extrapolated the overlap joint to 22 mm and maintained the same process conditions used for the samples with 12- and 16-mm overlaps. The variation in the adhesive strength achieved with this condition showed nonlinear growth (following a polynomial trend), as shown in Figure 9.

Equation (3) shows the second-degree polynomial regression equation obtained from the curve of the adhesive strength plotted as a function of the overlap, in a similar way as presented in previous equations:

Strength (N) = 449·Overval − 526.2·Overlap + 21.21·Overlap^2^, (3)

Through a DOE analysis, it was possible to analyze the behavior of adhesive strength for different process variables and joint overlap configurations. The adhesive strength between two materials is strongly affected by temperature, possibly because the interdiffusion mechanism is favored and is influenced by the injection flow rate. With a simplified model, it was possible to find that, for this specimen geometry, the process temperature and injection flow rate were decisively the most important process variables for obtaining good adhesion between dissimilar polymers with the same substrate profile.

## 4. Conclusions

The following conclusions were drawn by analyzing the adhesive strength of dissimilar materials obtained by overmolding. First of all, with the aid of the ANOVA experimental design, the effect of macrovariables from the injection process on the adhesive strength indicated that the interactions between temperature and injection flow rate were prominent. The combination of parameters that significantly affected the adhesive strength corresponded to a temperature of 260 °C and an injection flow rate of 90 cm^3^/s with an overlap of 16 mm. Also, the relationship between adhesive strength and overlap showed no linear growth. Simulation showed that the DOE can be an efficient way to quickly map processing parameter effects on adhesive strength obtained by multicomponent injection molding.

Studying polymer adhesion in multicomponent injection molding is a research area that offers many opportunities for innovation. The present methodology proposes the analyses of a plane deformation state by uniformly distributing the shear stress in a molded joint. The proposal to measure the adhesive strength by applying pure shear at the interface of the overmolded samples contributed significantly to reach the objectives of this study. For this, it was considered a production of sustainable overmolded composites up to 30% of natural fibers without additives.

## Figures and Tables

**Figure 1:**
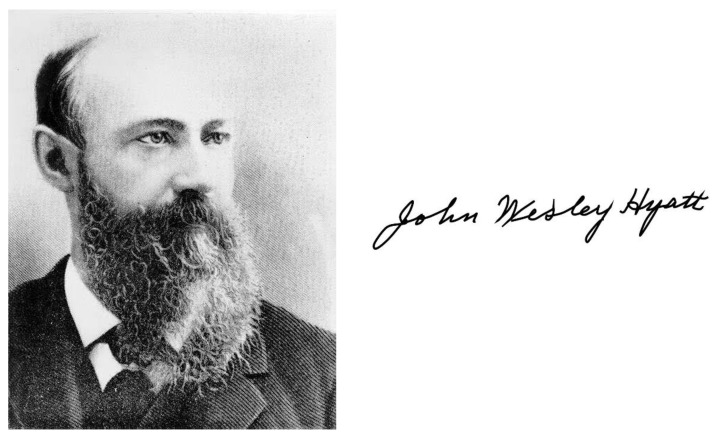
John Wesley Hyatt (1837–1920), American inventor of the first injection molding machine.

**Table 1:** Parameterized values of the injection molding process.

Process Variables	Injection Unit 1	Injection Unit 2 (L–H) *^a^*
Injection pressure (bar)	800	400
Holding pressure (bar)	400	(200–300)
Injection flow rate (cm^3^/s)	100	(30–90)
Injection temperature (°C)	210	(220–260)
Cooling time (s)	25	30

*^a^* L = low value; H = high value.

**Table 2:** Factor levels for the design of the polypropylene (PP)/composite factorial experiment.

Run	Holding Pressure (bar)	Injection Temperature (°C)	Overlap Length (mm)	Injection Flow Rate (cm^3^/s)
1	300	260	12	90
2	200	220	16	90
3	300	220	12	90
4	200	260	12	90
5	200	260	16	30
6	200	220	12	90
7	200	220	12	30
8	300	260	12	30
9	200	260	12	30
10	300	220	12	30
11	300	260	16	90
12	200	220	16	30
13	300	260	16	30
14	200	260	16	90
15	300	220	16	30
16	300	220	16	90

**Table 3:** Combined ANOVA for the 12- and 16-mm overlaps.

Source	DF	Adj SS	Adj MS	*F*-Value	*p*-Value
Model	15	2,147,656	143,177	62.16	0
Linear	4	1,841,923	460,481	199.9	0
HP (bar)	1	340	340	0.15	0.701
T (°C)	1	67,339	67,339	29.23	0
Fr (cm^3^/s)	1	105,438	105,438	45.77	0
O (mm)	1	1,668,805	1,668,805	724.45	0
2-Way Interactions	6	202,921	33,820	14.68	0
HP (bar)*T (°C)	1	37,813	37,813	16.42	0
HP (bar)* Fr (cm^3^/s)	1	19,857	19,857	8.62	0.004
HP (bar)*O (mm)	1	192	192	0.08	0.773
T (°C)* Fr (cm^3^/s)	1	90,014	90,014	39.08	0
T (°C)*O (mm)	1	9905	9905	4.3	0.04
Fr (cm^3^/s)*O (mm)	1	45,139	45,139	19.6	0
3-Way Interactions	4	100,071	25,018	10.86	0
HP (bar)*T (°C)* Fr (cm^3^/s)	1	46	46	0.02	0.888
HP (bar)*T (°C)*O (mm)	1	37,202	37,202	16.15	0
HP (bar)* Fr (cm^3^/s)*O (mm)	1	17,404	17,404	7.56	0.007
T (°C)* Fr (cm^3^/s)*O (mm)	1	45,419	45,419	19.72	0
4-Way Interactions	1	2742	2742	1.19	0.278
HP (bar)*T (°C)*Fr (cm^3^/s)*O (mm)	1	2742	2742	1.19	0.278
Error	112	257,997	2178		
Total	127	2,405,653			

Legend: DF—degrees of freedom; HP—holding pressure (bar); T—process temperature adjusted in plastification cylinder 2 (°C); Fr—injection flow rate (cm^3^/s); O—overlap.

## Data Availability

The data presented in this study are available on request from the corresponding author.
